# Lutembacher syndrome presenting as heart failure in an old female from rural Nepal: a case report

**DOI:** 10.1097/MS9.0000000000001873

**Published:** 2024-02-28

**Authors:** Durgesh Chaudhary, Pratik Adhikari, Binod Mehta, Pramodman Singh Yadav, Sagar Koirala, Sumit Kumar Shah, Manita Khadka

**Affiliations:** a Koirala Institute of Health Sciences, Dharan; b National Academy of Medical Sciences; c Nepal Medical College, Kathmandu, Nepal

**Keywords:** atrial septal defect, case report, heart failure, lutembacher syndrome, mitral stenosis

## Abstract

**Introduction and significance:**

Lutembacher syndrome (LS), combining atrial septal defect (ASD) and mitral stenosis (MS), is rare, particularly in rural areas. This case presents a 55-year-old Nepalese woman with LS symptoms; however, financial constraints hindered surgical treatment, highlighting LS challenges and the need for early intervention in resource-limited settings.

**Case presentation:**

A 55-year-old woman from rural Nepal presented with 30-day leg swelling and shortness of breath. Apart from autosomal dominant polycystic kidney disease (ADPKD) and smoking, she had no significant comorbidities. Clinical examination revealed severe mitral stenosis and an ASD, but financial limitations prevented surgery.

**Clinical discussion:**

LS is rarer in regions with low rheumatic heart disease (RHD) prevalence like Nepal. This case, despite rarity, delayed presentation, and financial barriers, emphasizes early intervention’s importance. While rheumatic fever wasn’t confirmed, clinical and echocardiographic findings suggest rheumatic mitral stenosis. The patient’s surgery reluctance due to finances highlights resource limitations’ impact.

**Conclusion:**

This Nepalese LS case highlights its complexity and management challenges, especially in resource-limited settings. It stresses early intervention’s importance and the impact of financial constraints on patient care. The study urges improved healthcare access and alternative funding in high RHD-prevalence regions.

## Introduction

Highlights

- A rare case of Lutembacher syndrome (LS) in a 55-year-old Nepalese woman presenting with heart failure.
- LS combines atrial septal defect (ASD) and acquired mitral stenosis (MS).
- Delayed diagnosis due to limited resources in rural setting.
- Despite severe cardiac issues, financial constraints impede surgical intervention.
- Emphasizes the urgent need for early diagnosis, timely intervention, and adequate funding for complex cardiac conditions like LS.

Lutembacher syndrome (LS) is an uncommon medical condition that manifests as a combination of ostium secundum atrial septal defect (ASD) that can be either congenital or acquired, along with congenital or acquired mitral stenosis (MS)^1^. In adherence to the SCARE (Surgical CAse REport) guidelines, we acknowledge a pivotal study by Alam and Khaled, titled “An incidental diagnosis of rheumatic mitral stenosis and secundum atrial septal defect (Lutembacher’s syndrome) in a young woman,” published in Case Reports in Cardiology in 2019. This study accentuates the extraordinary rarity of the condition in question, underscoring that “the occurrence of this combination of syndromes is exceedingly rare, with only one individual identified among a population of one billion people.” The historical origins of LS trace back to Johann Friedrich Meckel, an illustrious anatomist who first identified it in 1750. Nevertheless, it wasn’t until 1916 that Lutembacher provided a comprehensive characterization of this syndrome^2,3^. While the precise prevalence of LS remains elusive, its incidence may be more notable in regions where rheumatic heart disease (RHD) is prevalent, contingent upon the consistency of congenital ASD incidence. Sub-Saharan Africa bears a significant burden of RHD, affecting 15–20 cases per 1000 individuals. However, it’s noteworthy that only a limited number of LS cases have been formally documented within this region^4^. LS can manifest at any stage of life but is frequently observed in young women^5^.

In the subsequent sections of this report, we present a captivating case of LS involving a 55-year-old female from rural Nepal. Her initial medical presentation was prompted by bilateral leg swelling, an ailment that inadvertently led to the serendipitous discovery of this exceptionally rare syndrome.

## Case presentation

A 55-year-old woman arrived at the Emergency Department (ED) with complaints of bilateral leg swelling for 30 days. The patient also complains of shortness of breath during exertion (Grade II). The patient does not have any significant past medical history of diabetes mellitus, hypertension, pulmonary tuberculosis, or COPD. However, she was diagnosed with ADPKD in childhood, for which she was on medication for many years. Until the age of 18, she underwent conservative management for autosomal dominant polycystic kidney disease (ADPKD), which primarily involved adhering to a low-salt diet and ensuring adequate fluid intake. However, upon reaching the age of 18, she was prescribed Enalapril at an initial dose of 2.5 mg once daily (qd), which she diligently continued to take as part of her regular medication regimen. She has a personal history of smoking since childhood, with 4–5 cigarettes per day.

The patient complained of bilateral leg swelling, with the right leg exhibiting a noticeably greater degree of swelling compared to the left. Measurement revealed that the right leg had ~2 cm more swelling than the left. The patient noticed gradual or sudden swelling in the legs, particularly in the ankles and feet, which later progressed to the abdomen, face, and whole body. The swelling may be more pronounced at the end of the day and may improve after a night’s rest with the legs elevated. The swelling worsened during activities such as prolonged standing or sitting and was relieved by raising the legs. Shortness of breath often accompanied the swelling of the leg, especially while moving around or lying flat. The patient also experienced fatigue and weakness.

Furthermore, the patient had been experiencing Grade II exertional dyspnoea, which is characterized by shortness of breath that occurs during moderate physical activities, like climbing stairs or walking on level ground. However, it had progressed to the point where she also experienced shortness of breath at rest, especially when lying flat (orthopnea). The patient also gives a history of sudden episodes of breathlessness during the night (paroxysmal nocturnal dyspnoea). Upon initial assessment in the ED, her vital signs were as follows: a blood pressure of 140/80 mm Hg, a pulse rate of 70 beats per min, which was regular, a respiratory rate of 18 breaths per minute, a temperature of 98.4 degrees Fahrenheit, and an oxygen saturation of 96%. During the general examination, it was observed that the patient was conscious. She had no pallor, icterus, cyanosis, or clubbing except pitting oedema, which was present on bilateral legs; it was more prominent on the right leg. She had no pallor, icterus, cyanosis, or clubbing. However, on cardiovascular examination, we noticed raised jugular vein pressure (9 cm above the sternal angle). Upon precordial examination, we observed a normal apex beat in the left 5th intercostal space, albeit with a slight displacement of 1 cm lateral to the midclavicular line. There was a palpable P2 and left parasternal heave, but no palpable thrill was noted. The intensity of the first heart sound was loud, with a diastolic rumbling murmur best heard at the apex. Furthermore, an ejection systolic murmur was audible over the pulmonary area without any radiation or variation in respiration. Respiratory examination revealed bilateral equal air entry without any crepitations or wheezing. Abdominal examination revealed mildly distended with shifting dullness and hepatomegaly.

All the patient’s history and examination findings were consistent with right-sided heart failure which included jugular venous distention, hepatomegaly, and peripheral oedema. In addition, the patient experiencing grade II shortness of breath (SOB) upon exertion, was indicative of her cardiac condition. For the management of her heart failure, the patient was prescribed furosemide at an initial dose of 20–40 mg, administered intravenously (IV) once a day.

**Figure 1:**
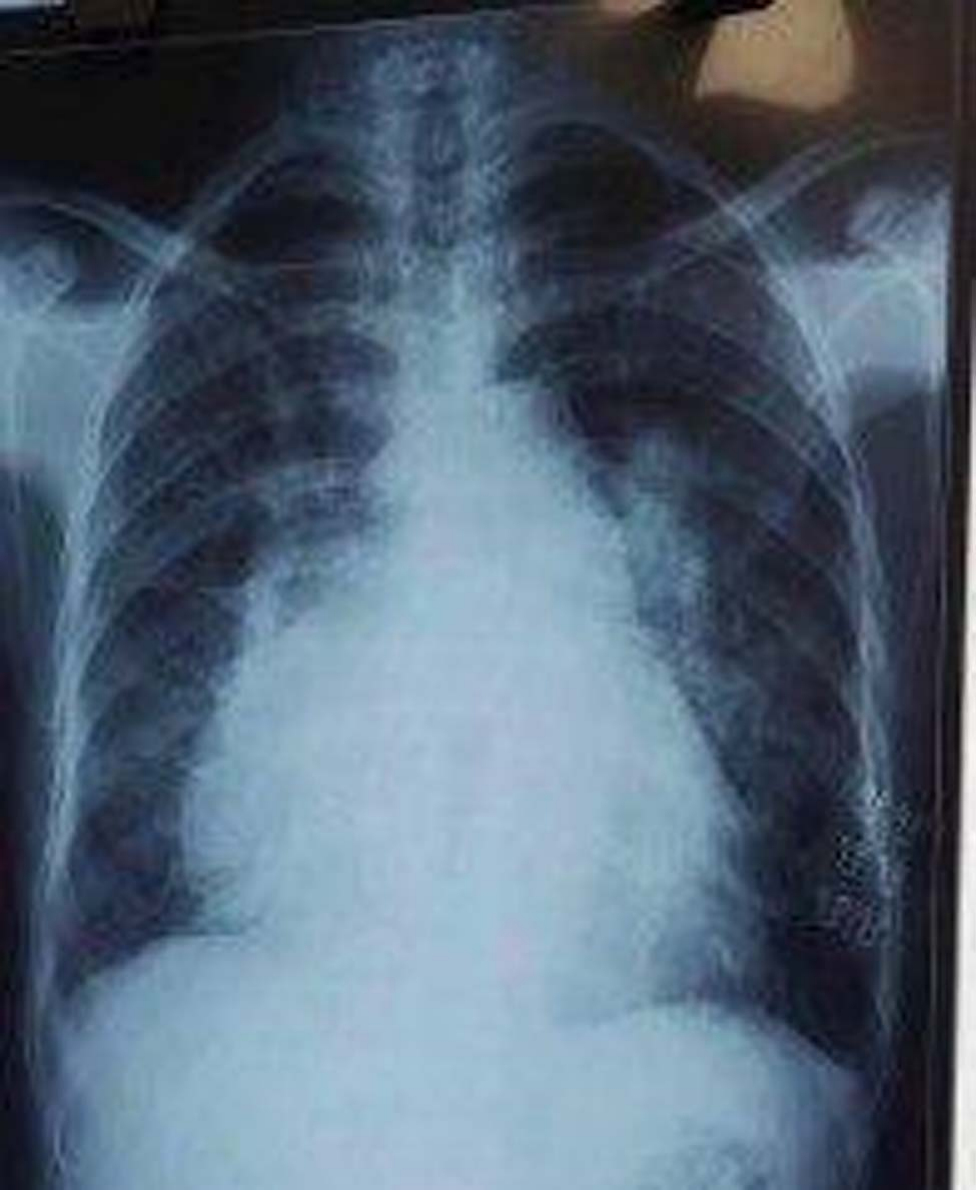
Chest X-ray showing bulging pulmonary conus, cardiomegaly with a cardiothoracic ratio of 0.7, and pulmonary plethora.

**Figure 2:**
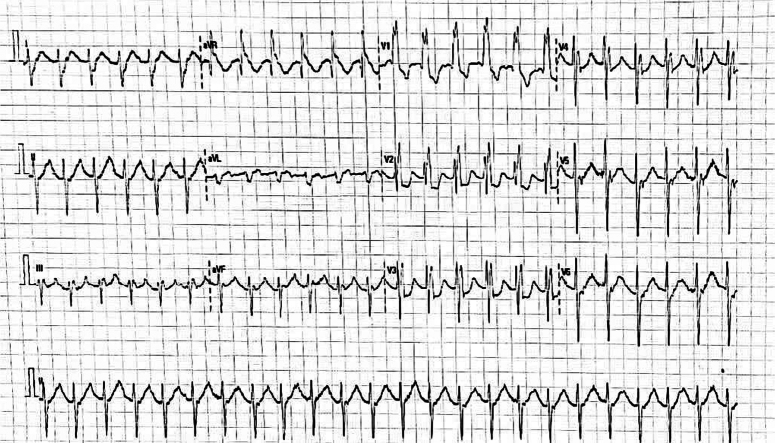
Electrocardiogram showing right bundle branch block with right axis deviation.

**Figure 3:**
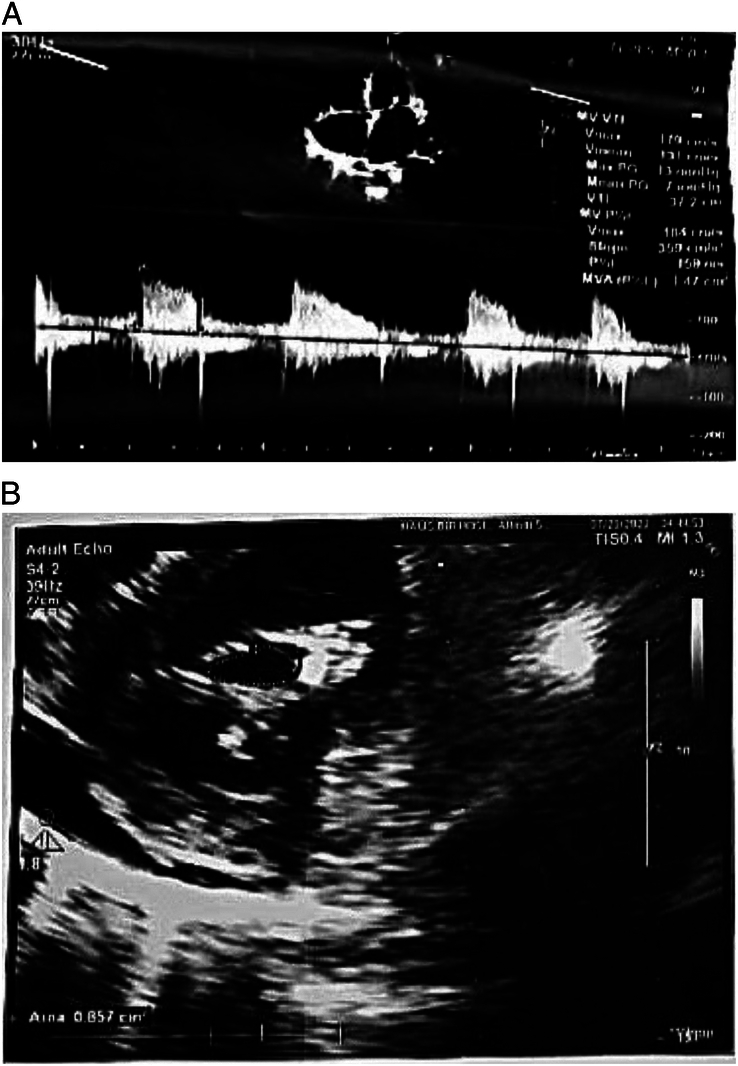
(A) Echocardiography findings with a 4-chamber view showing both mitral stenosis and atrial septal defect. (B) Echocardiography findings of parasternal short axis views showing stenosed mitral valve.

Her WBC count was 4600 cells/µl, her haemoglobin was 14.7 g/dl, and her platelets were 201 000 cells/µl. Urea and creatinine were 42 mg/dl and 1.2 mg/dl, respectively. Her sodium, potassium, and LFT were normal. A chest X-ray was ordered, which showed a bulging pulmonary conus, cardiomegaly with a cardiothoracic ratio (CTR) of 0.7, and a pulmonary plethora (Figure 1). The X-ray showed signs of lung congestion, but this congestion is not caused by pressure from the left atrium and pulmonary veins, which is typical in conditions like mitral stenosis. Instead, it’s because the right ventricle is being filled too much. The ECG showed a right bundle branch block with right axis deviation (Figure 2). Subsequently, her echocardiogram was done, which depicted a hugely dilated right atrium (RA), right ventricle (RV), and left atrium (LA) measuring 5 cm in dimension with a restricted opening of the mitral valve (Figure 3A, B). Mitral valve planimetry showed the mitral valve area (MVA) was 0.8 cm^2^. There was LV Systolic dysfunction (LVEF: 40%), moderate to severe TR, and moderate PAH with a PASP of 52 mm Hg. There was also an ASD secundum measuring 1.1 cm with a left-to-right shunt. The patient had severe mitral stenosis and ASD secundum; therefore, intervention is required to prevent rising pulmonary artery pressure by increasing flow through the right-sided heart, and she had occasional shortness of breath on exertion as well. The patient rejected treatment due to financial constraints. The patient was then discharged on furosemide (20 mg) upon reduction of oedema. I am writing in accordance with the SCARE checklist. In accordance with the SCARE 2023 guideline (Sohrabi *et al.*, 2023), the methodology for reporting surgical case details was strictly adhered to in this study^17^.

## Discussion

The pattern of prevalence of congenital heart disease (CHD) reported in Nepal varies according to region. In a systematic review and meta-analysis study conducted by Shrestha and colleagues from 2002 to 2022, the prevalence of congenital heart disease in Nepal was 0.7%, and the frequency of male patients was higher. The atrial septal defect was the commonest on the whole, while the tetralogy of Fallot was the commonest among the cyanotic variety^6^. There is no specific data available on the prevalence of Lutembacher syndrome in Nepal. It is generally considered to be a rare condition worldwide. Since the prevalence of Lutembacher syndrome is low globally, it is likely to be uncommon in Nepal as well. However, it is likely to be prevalent in this region due to the high prevalence of rheumatic heart disease.

Lutembacher syndrome is a rare clinical condition with an incidence of 0.001 per million people in the United States. The first case report was published by Rene Lutembacher in 1916, describing a 61-year-old woman with congenital ASD (secundum type) and congenital MS who had been pregnant seven times. Over time, other forms of LS have been documented, including acquired mitral stenosis (often rheumatic in origin, which is the most common form of MS observed in LS), acquired/iatrogenic ASD (usually resulting from trans-septal puncture during mitral valvuloplasty for acquired MS), and reverse LS (involving a right-to-left shunt ASD and tricuspid stenosis)^7^.

In low-income countries like Nepal, where the burden of rheumatic heart disease is relatively high, LS is commonly first diagnosed in symptomatic young adults^8,9^. Patients may present with various symptoms, such as fatigue, exercise intolerance, or overt signs of heart failure. In our case, the patient presented with heart failure symptoms in her fifth decade of life. While a past history of rheumatic fever could not be established from the clinical records (reported to be absent in 60% of LS patients)^10^, the patient’s age, the increased prevalence of rheumatic heart disease in the region, and echocardiographic findings showing significantly dilated right atrium (RA), right ventricle (RV), and left atrium (LA) with restricted opening of the mitral valve (Figure 3) led us to conclude that the mitral stenosis was of rheumatic origin, following the World Heart Federation criteria for diagnosing rheumatic heart disease^11^. Mitral valve planimetry revealed a mitral valve area (MVA) of 0.8 cm^2^ (Figure 3B). Additionally, there was moderate to severe tricuspid regurgitation (TR) and moderate pulmonary arterial hypertension (PAH) with a pulmonary artery systolic pressure (PASP) of 52 mm Hg. A secundum ASD measuring 1.1 cm with a left-to-right shunt was also identified (Figure 3B). The ASD is believed to be congenital in origin, as there is no history of previous transcatheter intervention. The usual signs of pure MS, like a specific type of murmur, a loud first heart sound, a big left atrium, lung congestion, breathing difficulties, and coughing up blood, are not as common or quick to appear. This is because an opening in the heart (ASD) helps release pressure from the left atrium. This makes the right atrium and ventricle grow bigger and can cause heart failure on the right side. Percutaneous transcatheter therapy has become the preferred non-surgical approach for managing Lutembacher syndrome, with fewer complications, provided there are no contraindications to catheter closure of the ASD or catheter balloon mitral valvuloplasty^12^. Kukarni *et al.* ^13^ reported that LS presenting with heart failure and pulmonary hypertension are poor prognostic indicators, as seen in our patient. In general, it has been observed that a significant number of patients in Nepal, Nigeria, and the West African subregion present late to the hospital due to various reasons, including the initial use of traditional/herbal therapy and financial constraints in accessing conventional medicine^14–16^. Given her significant history of ADPKD, she had been compliant to her medications from an early age. Since her renal function test was normal the renal cause of swelling had been ruled out.

Our case report presents a complex and rare occurrence of LS in a Nepalese patient, shedding light on the intricate nature of this condition and the challenges it poses to management, especially in resource-limited settings. Notably, the patient’s electrocardiogram (ECG) findings revealed a right bundle branch block with right axis deviation, indicative of the impact of LS on the heart’s electrical conduction system. These ECG findings underscore the significance of comprehensive cardiac assessments in LS cases. Furthermore, our report underscores the urgency of early diagnosis and intervention in LS, emphasizing that delayed recognition can exacerbate symptoms and heighten risks. Financial constraints, a significant barrier to care, were regrettably a factor in this case. To address such challenges in resource-limited regions like rural Nepal, alternative funding options and collaborations with governmental and non-governmental organizations are imperative to ensure comprehensive care for individuals with complex cardiac conditions. In conclusion, our study not only advances the understanding of LS but underscores the vital need for equitable healthcare access and thorough evaluations, including ECG analysis, to enhance the prognosis of LS patients, especially in areas with a high prevalence of rheumatic heart disease. However, it’s important to acknowledge certain limitations in our case report. Firstly, due to financial constraints, the patient was unable to undergo the planned definitive surgical treatment for LS, highlighting a real-world challenge in resource-limited settings. Secondly, we couldn’t establish a definitive history of rheumatic fever, a common precursor of mitral stenosis in LS, which could impact the diagnostic accuracy. Thirdly, our report presents a single case, and while it sheds light on the complexity of LS in Nepal, it doesn’t provide a comprehensive epidemiological overview. Despite these limitations, our study underscores the urgent need for equitable healthcare access and early intervention in cases of complex cardiac conditions.

## Prognosis

In complex cases like this, prognosis remains uncertain, particularly when financial constraints limit essential interventions. Untreated mitral stenosis and ASD secundum can worsen symptoms, impair exercise tolerance, and heighten heart failure risks. Progressive dyspnoea during exertion and nocturnal episodes signals increasing cardiac strain. Access to timely care is critical, emphasizing the need for equitable healthcare access for individuals with complex cardiac conditions.

## Conclusion

The case report underscores the rarity and complexity of LS in a Nepalese patient, particularly in resource-limited settings. The patient’s presentation with heart failure symptoms at a later stage of life highlights the challenges of delayed diagnosis and intervention in rural regions. The coexistence of congenital ASD and acquired MS in LS poses clinical intricacies. Financial constraints emerged as a significant barrier to accessing appropriate care, illustrating the impact of resource limitations on patient outcomes. The case emphasizes the importance of early diagnosis, intervention, and exploring alternative funding options to address such challenges. The study reinforces the need for heightened awareness, timely management, and collaborative efforts to improve the prognosis of LS patients, especially in regions with high prevalence of rheumatic heart disease.

## Ethical approval

Not applicable.

## Consent

Written informed consent was obtained from the patient for publication of this case report and accompanying images. A copy of the written consent is available for review by the Editor-in-Chief of this journal on request.

## Source of funding

None.

## Author contribution

B.M.: resources, conceptualization. D.C.: writing: original draft of manuscript, review and editing. P.A.: writing: original draft of manuscript, review and editing, supervision. P.S.Y.: resources, writing: review and editing. S.K.S.: writing: review and editing. M.K.: writing: review and editing. S.K.: writing: review and editing.

## Conflicts of interest disclosure

The author declared no relevant financial conflict or any other conflict of interest.

## Research registration unique identifying number (UIN)

This is a case report involving a human subject, so registration of research study was done.

1. Registry used: Researchregistry.com.

2. Unique Identifying number or registration ID: researchregistry9390.

## Guarantor

Pratik Adhikari.

## Data availability statement

The datasets supporting the conclusions of this article are included within the article.

## Provenance and peer review

Non commissioned, externally peer-reviewed.
